# Genotype by sequencing identifies natural selection as a driver of intraspecific divergence in Atlantic populations of the high dispersal marine invertebrate, *Macoma petalum*

**DOI:** 10.1002/ece3.3332

**Published:** 2017-09-03

**Authors:** Stacy L. Metivier, Jin‐Hong Kim, Jason A. Addison

**Affiliations:** ^1^ Department of Biology University of New Brunswick Fredericton NB Canada

**Keywords:** high dispersal, marine invertebrate, natural selection, outlier, population genomics, single nucleotide polymorphism

## Abstract

Mitochondrial DNA analyses indicate that the Bay of Fundy population of the intertidal tellinid bivalve *Macoma petalum* is genetically divergent from coastal populations in the Gulf of Maine and Nova Scotia. To further examine the evolutionary forces driving this genetic break, we performed double digest genotype by sequencing (GBS) to survey the nuclear genome for evidence of both neutral and selective processes shaping this pattern. The resulting reads were mapped to a partial transcriptome of its sister species, *M. balthica*, to identify single nucleotide polymorphisms (SNPs) in protein‐coding genes. Population assignment tests, principle components analyses, analysis of molecular variance, and outlier tests all support differentiation between the Bay of Fundy genotype and the genotypes of the Gulf of Maine, Gulf of St. Lawrence, and Nova Scotia. Although both neutral and non‐neutral patterns of genetic subdivision were significant, genetic structure among the regions was nearly 20 times higher for loci putatively under selection, suggesting a strong role for natural selection as a driver of genetic diversity in this species. Genetic differences were the greatest between the Bay of Fundy and all other population samples, and some outlier proteins were involved in immunity‐related processes. Our results suggest that in combination with limited gene flow across the mouth of the Bay of Fundy, local adaptation is an important driver of intraspecific genetic variation in this marine species with high dispersal potential.

## 1 INTRODUCTION

Historic disturbances play a major role in shaping the population structure of marine invertebrates (Grantham, Eckert, & Shanks, 2003). During the last glacial maximum (LGM), which occurred 19–23 kyr before present, the Laurentide ice sheet covered North America down to its southern limit at Cape Cod. Sea levels at this time were approximately 120–130 m lower than present (Huybrechts, 2002; Peltier, 2002; Yokoyama, Lambeck, De Deckker, Johnston, & Fifield, 2000). Therefore, along the Atlantic coast of North America, many marine species were displaced not only by ice and lower temperatures in northern ranges (Dyke et al., 2002; Maggs et al., 2008), but also by an overall lower sea level which resulted in North Atlantic coastlines retreating seaward (Maggs et al., 2008).

The glacial cycles during the Pleistocene resulted in major range changes, local extirpations, and the establishment of refugial populations for many species of coastal marine invertebrates in the North Atlantic (Hewitt, 1996, 2000). For species that did not exclusively survive in North American refugia, recolonization events from Europe have influenced, in part, the contemporary species composition of the intertidal section of the Atlantic coast following the LGM, especially for obligate rocky shore species (Ingolfsson, 1992; Wares & Cunningham, 2001). In contrast, species found on soft substrate have a higher level of endemism on the northwestern Atlantic coast, which indicates that these species likely survived the LGM in refugia (Cronin, 1988; Ingolfsson, 1992). The main southern refugial areas for northwestern Atlantic species occurred along the Carolinas, south into Florida and the Gulf of Mexico for marine taxa, with northern refugia suggested for some species (Maggs et al., 2008; Marko, 2004; Wares & Cunningham, 2001; Young, Torres, Mack, & Cunningham, 2002). One or more refugia have been proposed within the Atlantic Canadian coastline itself, including genetic evidence for a Nova Scotia refugium (Bernatchez, 1997; Maggs et al., 2008; Young et al., 2002). For species surviving in southern refugia, postglacial recolonization is characterized by expansion of a subset of the southern genotypes, resulting in less diversity in the newly colonized northern populations (Hewitt, 1996, 2000; Maggs et al., 2008). However, such genetic differentiation is not expected to be directly correlated with distance between populations in marine environments as a result of dispersal abilities, including planktonic larvae and rafting of marine invertebrates, or recent recolonization events (White et al., 2010).

Dispersal ability of species also plays an important role in determining population structure over time. Panmixia is predicted for species having long‐lasting planktonic larval stages as these larvae may disperse with surface currents, although the frequency of long‐distance dispersal is dependent on multiple factors, including the size of the parent population, the probability of larvae being carried offshore into the major ocean current systems, and larval mortality (Doherty, Planes, & Mather, 1995; Scheltema, 1971). In addition, dispersal distance depends on the length of time larvae spend in the plankton and the duration of time they are physiologically capable of settling (Siegel, Kinlan, Gaylord, & Gaines, 2003; Watson, Kendall, Siegel, & Mitarai, 2012). A direct correlation has been suggested between planktonic larval duration and larval displacement (Siegel et al., 2003; Waples, 1987). However, pelagic larval duration has been only weakly correlated to *F*_ST_ (Weersing & Toonen, 2009) likely due to active vertical migration in the water column, temporal variability, and oceanographic features such as mesoscale eddies, alongshore jets, and squirts (Watson et al., 2012; Weersing & Toonen, 2009; White et al., 2010). For species with nonplanktonic larval development, transportation of egg cases and juveniles by rafting on microalgal mats or ice appears to be common (Grantham et al., 2003; MacFarlane, Drolet, Barbeau, Hamilton, & Ollerhead, 2013; Marko, 2004).

**Figure 1:**
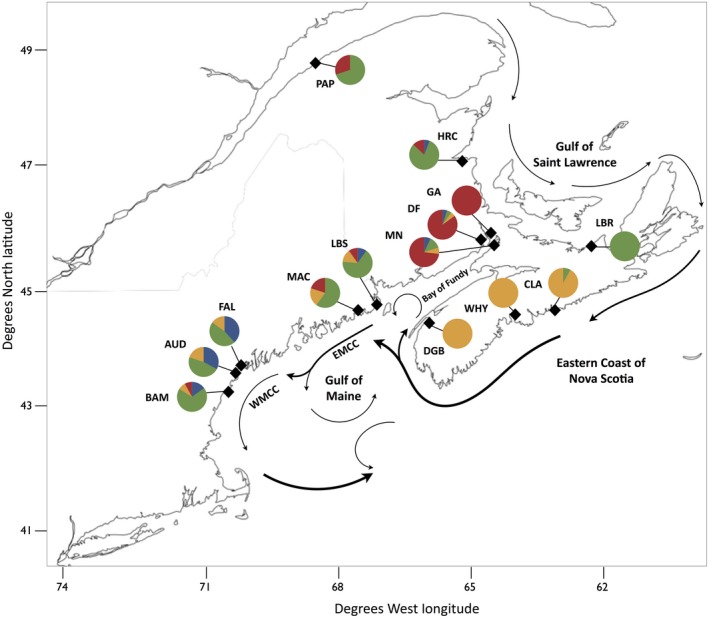
Map of sites sampled for *Macoma petalum* between May 2011 and July 2014 in four main regions: the Gulf of Saint Lawrence, Nova Scotia, the Bay of Fundy, and the Gulf of Maine (see Table 1 for abbreviations). Pie charts represent the frequency of mtDNA haplotypes (704‐bp *COI/COIII*,* n *=* *194) from each site belonging to the four main lineages described in Einfeldt et al. (2017). Eastern Maine Coastal Current (EMCC) and the Western Maine Coastal Current (WMCC) are indicated, and all current patterns are adapted from Aretxabaleta et al. (2008), Pettigrew et al. (2005), and Drinkwater and Gilbert (2004). Map data ©2016 Mapbox (www.mapbox.com/about/maps/)

The Bay of Fundy (BF) in the northwest Atlantic is an important phylogeographic break for at least two species of marine invertebrates lacking a pelagic larval stage, *Hediste diversicolor* and *Corophium volutator* (Einfeldt & Addison, 2013; Einfeldt, Doucet, & Addison, 2014), as well as two species of planktonic dispersers, *Macoma petalum* and *Tritia obsoleta* (Einfeldt, Zhou, & Addison, 2017). Restricted gene flow across this boundary provides the opportunity for populations to diverge as a result of both genetic drift and natural selection. The Gulf of Maine (GOM) coastal currents flow in a counterclockwise circular pattern, impeding the mixing of waters in the Gulf of Maine and the Bay of Fundy (Pettigrew et al., 2005; White et al., 2010; Figure 1). However, reversals of flow direction caused by westerly winds are relatively common in the coastal zone, and can spread egg and larval drift in the opposite direction (Brickman, 2014). Patterns of mtDNA variation in all four species studied to date suggest a strong role for limited gene flow and genetic drift in maintaining the genetic break across the mouth of the Bay of Fundy.

In addition to neutral processes driving genetic differences among populations, the genetic split at the Bay of Fundy could also result from local adaptations caused by natural selection. Locally, Chu, Kaluziak, Trussell, and Vollmer (2014) identified putative selection for heat stress‐mediating genes using next‐generation sequencing (NGS) techniques based on thermal tolerance differences in *Nucella lapillus*, a species lacking a planktonic life stage. They found relatively high levels of gene flow, likely due to rafting in this species, but also genetic splits between the East and West Gulf of Maine Coastal Currents and Nova Scotia. *Semibalanus balanoides*, an intertidal barnacle with pelagic larvae, was found to exhibit genetic structure both between the Gulf of Maine and the Gulf of Saint Lawrence (GSL) and within the GSL, and this differentiation within the GSL was attributed to the potential effects of selection due to temperature differences (Holm & Bourget, 1994). Therefore, limited gene flow across the Bay of Fundy/Gulf of Maine biogeographic break may promote the accumulation of genetic divergence driven by natural selection and local adaptation in marine invertebrates.

*Macoma petalum* is a tellinid bivalve species endemic to the northwestern Atlantic coast and introduced to the northeast Pacific coast of North America (Nikula, Strelkov, & Vainola, 2007). It is infaunal, inhabiting soft‐bottom intertidal mudflats. *Macoma petalum* invaded the Atlantic coast during the mass trans‐arctic invasions of the middle Pliocene, about 3.5 mya, when the Bering Strait opened up in the North Atlantic (Kamermans, Van der Veer, Witte, & Adriaans, 1999; Meehan, Carlton, & Wenne, 1989; Nikula et al., 2007; Vainola, 2003). *Macoma petalum* has planktonic larvae, and in other *Macoma* spp., these larvae are known to survive up to 6 weeks (Jansson, Norkko, & Norkko, 2013). *Macoma petalum*'s long‐lived pelagic larvae, and thus high dispersal potential, lack of recolonization from Europe following the LGM, and endemism to the northwestern Atlantic, make it the ideal candidate to study the evolutionary mechanisms driving the biogeographic break at the Bay of Fundy.

We examined *M. petalum* from the Gulf of Maine (GOM), the Gulf of Saint Lawrence (GSL), the Bay of Fundy (BF), and Nova Scotia (NS) to study the roles of selection and genetic drift driving patterns of genetic differentiation between these regions. We aimed to build on previous works that used mtDNA and limited nuclear markers to investigate the phylogeographic break at the Bay of Fundy (Einfeldt & Addison, 2013; Einfeldt et al., 2014, 2017). Our main goal was to determine whether this genetic break was primarily a result of limited gene flow and random genetic drift, or whether differential selective pressures within the Bay of Fundy were responsible for driving genetic change. As previous research employed putatively neutral mtDNA variation, we similarly expect small but significant differentiation across the nuclear genome as a result of limited gene flow and genetic drift throughout the region. In contrast, if populations of *M. petalum* are locally adapted to the mudflats in the Bay of Fundy, then we expect to detect a handful of loci with signatures of strong differentiation between regions. By assessing the levels of genome wide genetic structure, our research will provide insight into the importance of both dispersal limitation and local adaptation to the formation of the biogeographic break observed at the community level across the mouth of the Bay of Fundy.

## 2 METHODS

### 2.1 Preparing and assembling transcriptome reference

Pooled reads from the *M. balthica* transcriptome were downloaded from the NCBI SRA database (Accession SRX145744‐6; Pante et al., 2012) and imported into CLC Genomic Workbench 8.0.3 (CLC Bio). We trimmed the raw reads for a maximum of two ambiguous nucleotides, a minimum PHRED score of 20, and a minimum length of 50 bp. Additionally, we trimmed out short population‐specific 5′ repeats and adapters from the original MINT cDNA Synthesis Kit (Evrogen) used to make the cDNA library.

Trimmed reads were assembled in CLC 8.0.3 with default settings, and were remapped with a length fraction of 0.5 and a similarity fraction of 0.8 as these criteria yielded the most contigs with two or more contributing reads. We used less stringent parameters than Pante et al. (2012) in creating the transcriptome reference to avoid excluding informative SNPs due to genetic differences between *M. balthica* and *M. petalum*. We kept contigs with a minimum of two contributing reads and retained high‐quality singletons.

We filtered out duplicate contigs in CLC 8.0.3 by performing a BLASTn search of the transcriptome assembly against itself. Sequences were discarded if they were a perfect match to another, longer sequence. We retained only representative contigs and singletons to create a partial reference transcriptome for analysis of Illumina reads.

### 2.2 DNA extraction and library preparation

*Macoma petalum* individuals were sampled from 14 intertidal sites in the Gulf of Maine, the Bay of Fundy, Nova Scotia, and the Gulf of Saint Lawrence between May 2011 and July 2014 (Figure 1). Individual tissue samples were preserved in 95% ethanol and stored at −20°C until DNA extraction.

We extracted genomic DNA from all 60 individuals following the DNeasy Blood & Tissue Kit (QIAGEN) protocol. We added 10 μl RNase A to the extraction homogenate and incubated overnight at 56°C. Genomic DNA from each individual was eluted in 100 μl of EB buffer. We checked the quality of extracted DNA by running an aliquot on a 1% agarose gel for UV imaging and measuring concentration with the QUIBIT 2.0 Fluorometer Broad Range assay (Invitrogen). Mitochondrial sequences from previous analysis of the same individuals were used to confirm that all individuals were indeed *M. petalum* and not *M. balthica* as *M. balthica*'s range extends into the Gulf of Saint Lawrence (Einfeldt et al., 2017).

### 2.3 Library construction and purification

We digested 100 ng of genomic DNA from each individual with two restriction enzymes, 5 U/μl of Sau96I and 10 U/μl of MluCI (BioLabs) with 1× buffer in a 20 μl reaction. Digestion was completed in a thermocycler at 37°C (2 hr), 80°C (20 min), and 4°C (hold). Barcoded Illumina adapters were ligated to digested DNA at 22°C (2 hr), 65°C (30 min), and 4°C (hold). We used 60 unique barcodes (0.0125 μmol/L) of 4–10 bp to avoid sequencing bias during Illumina calibration, along with four barcodes on the Y‐adapter (1.25 μmol/L), one barcode per 15 individuals, between 5 and 8 bp in length. Reaction components additionally included 1× buffer, 1 μmol/L of ATP, and 400 U of T4 ligase in a 40 μl reaction. We multiplexed individual DNA into two libraries to maximize PCR amplification using 10 μl of ligated DNA from each individual, with 32 individuals in the first library and 28 individuals in the second. The pooled ligates were purified using the QIAquick PCR Purification Kit (QIAGEN), with 50 μl eluate, prior to amplification to remove adapter and enzyme contamination. We followed the NEBNext High‐Fidelity 2× PCR Master Mix (Biolabs) protocol for PCR in 5 × 40 μl reactions per pool. Thermal cycling conditions were 98°C (30 s) initial denaturation, 98°C (10 s), 65°C (30 s), and 72°C (30 s) repeated for 16 cycles, 72°C (5 min) final extension, and 4°C (hold).

Four of the five 40 μl PCRs were pooled for each library prior to an additional purification using the QIAquick PCR Purification Kit (QIAGEN), with a final elution volume of 25 μl per library. The remaining PCR product for each pool was used to verify success of PCR in a 1.5% agarose gel and to determine the concentration of DNA after purification.

We ran the two pooled libraries of PCR products in a 1.5% agarose gel (75 V, 1 hr) for a gel extraction. We used the section of the gel between 200 and 700 bp to ensure removal of nonspecific bands <200 bp in size. The QIAEX II Gel Extraction Kit was used to purify the gel sections, finally eluting 30 μl of each pool and combining for a total of 60 μl for the final library. We sent 50 μl of the library to the McGill University and Génome Québec Innovation Centre for 100‐bp paired‐end sequencing in a single lane of the Illumina HiSeq 2000 PE100 platform.

### 2.4 Processing raw Illumina reads

Raw Illumina reads were downloaded from the Nanuq portal and imported into CLC 8.0.3. We demultiplexed the reads based on Y‐adapter and unique barcodes, discarding reads lacking barcodes. Reads were trimmed for a minimum PHRED score of 30, a limit of two ambiguous nucleotides, and for a minimum length of 30 bp. Reads were then mapped to our partial *M. balthica* transcriptome reference using the CLC Assembly Cell 4.3 algorithm with default parameters, a length fraction of 0.9, and similarity fraction of 0.8. A consensus sequence was extracted from this mapping, with any unmapped regions filled in by the transcriptome reference assembly, and used as the reference for all future bioinformatics analyses (e.g., variant detection and BLASTX to identify outlier loci). Variants were detected for each individual and for the reference as a whole using CLC's Basic Variant detection tool. All variants except single nucleotide variants (SNVs) were excluded, and only those SNVs that had at least two reads of coverage for each of the 60 individuals were kept. Therefore, only biallelic SNPs with coverage on the transcriptome reference as well as in all individuals were kept for future use. SNPs were filtered for a minor allele frequency of ≥0.05 to obtain a final SNP dataset for use in future analysis.

### 2.5 Summary statistics

The files exported from CLC were processed and converted to necessary file types for further analysis using PDGSpider 2.0.8.3 (Lischer & Excoffier, 2012). We used Arlequin 3.5.2.2 (Excoffier & Lischer, 2010) to calculate the number of polymorphic loci (*P*), expected heterozygosity (*H*_e_), and observed heterozygosity (*H*_o_) at all sampling sites as well as pairwise *F*_ST_ values between each sampling site. We calculated locus‐specific *F*_ST_ values in Genepop 4.5.1 (Rousset, 2008).

### 2.6 Outlier detection

We used BayeScan v2.1 (Foll & Gaggiotti, 2008) and LOSITAN (Antao, Lopes, Lopes, Beja‐Pereira, & Luikart, 2008), which implements fdist (Beaumont & Nichols, 1996), to detect loci putatively under selection. Both BayeScan and LOSITAN use *F*_ST_‐based outlier detection methods. LOSITAN identifies outlier loci as those that have a significantly different *F*_ST_ than expected for the neutral model for a given heterozygosity. BayeScan partitions locus–population *F*_ST_ coefficients into a population‐specific component shared by all loci and a locus‐specific component shared by all populations. Outliers are those loci that require the locus‐specific component to explain observed genetic diversity. BayeScan was run using default settings, meaning a sample size of 5,000, thinning interval of 10, 20 pilot runs of length 5,000, and an additional burn‐in of 50,000, resulting in a total of 100,000 iterations following the initial burn‐in. We filtered loci by a *q* value of 0.10 and repeated the runs three times to ensure consistency. Runs in LOSITAN were performed using the neutral mean *F*_ST_, which performs an initial run with all SNPs, removes potential outliers to recalculate a more accurate neutral mean *F*_ST_, and then uses this neutral *F*_ST_ for the actual analysis. The mean *F*_ST_ was also forced with a confidence interval of 0.995 and false discovery rate (FDR) of 0.1. We ran 1 million simulations with the infinite allele mutation model. Analyses were repeated 10 times and only outliers present in all 10 runs were retained. For both LOSITAN and BayeScan, runs were performed with data divided into the two sampling regions based on the assignment tests using STRUCTURE (see Results): the Bay of Fundy or the coastal populations from the Gulf of Saint Lawrence, Nova Scotia, and the Gulf of Maine regions. To create a neutral dataset, all detected outliers were filtered out of the final SNP dataset, and only one SNP was kept per contig to minimize the effects of linkage disequilibrium. We used BLASTX in Blast2GO Basic 3.1.12 to determine the identity of outlier contigs and singletons with an e‐value threshold of 10^−3^ and default settings in the eukaryote NR protein database.

### 2.7 Assessing population structure

We tested for population structure using the program STRUCTURE 2.3.4 (Falush, Stephens, & Pritchard, 2003; Pritchard, Stephens, & Donnelly, 2000). STRUCTURE probabilistically assigns individuals to *k* detected clusters based on their allele frequencies. The program was independently run on the total SNP dataset and the dataset containing only putatively neutral unlinked SNPs. Simulations were run with the admixture model, correlated allele frequencies, a burn‐in period of 50,000 and 500,000 MCMC repetitions for 1 < *k *<* *5 with five iterations for the full dataset and the neutral dataset, and 10 iterations for the outlier datasets. The Evanno, Regnaut, and Goudet (2005) method was implemented in STRUCTURE HARVESTER (Web 0.6.94; Earl & vonHoldt, 2012) to infer the *k* clusters that best fit the data.

We also performed an analysis of molecular variance (AMOVA) in Arlequin 3.5.2.2 with 10,000 permutations using the full SNP dataset, the outlier dataset, and the neutral dataset to partition variance among regions, among populations within regions, and within populations. Populations were assigned to one of the two regions indicated by the structure analysis.

We performed a principle components analysis (PCA) to visualize population structure at a greater resolution. PCA reduces the genetic variation of multivariate data into principle components, which are then visualized in a plot to infer genetic relationships between individuals. We used the adegenet 2.0.0 package (Jombart, 2008; Jombart & Ahmed, 2011) in *R* (version *3.3*.2; R Core Team 2016) to perform the analysis with the dudi.pca algorithm. PCA was run on the combined SNP dataset, the neutral dataset, and the outlier loci datasets.

## 3 RESULTS

### 3.1 Assembly and descriptive NGS results

We downloaded 450.1 mol/L bases over 871,962 cleaned reads of *M. balthica* transcriptome from the NCBI SRA database (Accession SRX145744‐6; Pante et al., 2012). After trimming for quality, length, and adapter contamination, 76.3 mol/L bases remained over 241,194 reads, resulting in 27.66% of the raw reads and 16.95% of raw bases being retained for the assembly process.

The assembled *M. balthica* partial transcriptome reference yielded 9,795 contigs, with 60.70% of reads remapped. The N50 statistic for these contigs was 633, meaning that 50% of the bases were found on contigs equal to or >633 bp in length. After filtering for duplicate or highly similar sequences, 9,299 contigs were retained. From this assembly, 90,600 high‐quality singletons were also retained and used with the contigs to create the partial transcriptome reference.

The partial *M. balthica* transcriptome assembled by Pante et al. (2012) consisted of 1,714 total contigs (1.96 mol/L bases) following stringent quality filtering, including removal of all singletons, minimum contig length of 400 bp, and base call quality ≥42. The current assembly retained more contigs and bases than Pante et al. (2012) despite additionally filtering adapters from the MINT cDNA synthesis kit (Evrogen) as reads were trimmed with a lower‐quality threshold (PHRED score 20) and a shorter minimum length (50 bp) to retain variation for mapping *M. petalum* reads.

Illumina sequencing of *M. petalum* genomic DNA yielded 309.1 million paired‐end reads. These raw reads were demultiplexed by barcode, resulting in an average of 4.860 million reads per individual and an average read length of 89.36 bp. After quality trimming for PHRED score and minimum length, a total number of 22.03 billion bases remained over 261.2 million reads at an average of 4.353 million reads per individual of with an average length of 83.56 bp per read. We mapped 85.92 million reads (7.272 billion bases), 32.90% of the processed reads, to the *M. balthica* reference to extract a consensus sequence. Finally, 36.85% of processed Illumina reads (96.26 million reads, 8.126 billion bases) were mapped to the consensus reference, resulting in 24,163 contigs and singletons with at least two contributing reads from each individual. Only variants that mapped to the *M. balthica* reference transcriptome were included in further analysis.

We detected a total of 1.199 million variable sites in the reference sequence, 454,264 of which had at least two contributing reads, including indels, replacements, and multinucleotide variants. We filtered this down to 14,310 SNPs with a minimum of two reads of coverage per individual, and the average depth of the heterozygous SNPs (no. of heterozygous SNP‐detected reads/no. of heterozygous SNPs; Table S1) ranged from 19.59 to 35.64. The final SNP dataset consisted of 2,583 SNPs, scored in all individuals, on 1,415 contigs with a minor allele frequency >0.05.

### 3.2 Summary statistics

Summary statistics for each sampling site and region consisted of the proportion of polymorphic loci, observed heterozygosity, and expected heterozygosity averaged across all loci (Table 1). The proportion of polymorphic loci was highest in GSL and lowest in the GOM, with the BF and NS sites having similar average *P*. Expected and observed heterozygosity among the four regions are comparable. The average *H*_e_ and *H*_o_ per site decreased northward (although standard deviations are large) due a high proportion homozygous sites among individuals.

**Table 1:** Sampling distribution of *Macoma petalum* individuals for GBS library construction and estimates of genetic diversity (*P*: the percentage of polymorphic loci; *H*_e_: expected heterozygosity; *H*_o_: observed heterozygosity) based on 2,583 SNPs with minor allele frequency ≥0.5 calculated in Arlequin 3.5.2.2

Region	Site	*N*	*P* (%)	*H*_o_	*H*_e_
Gulf of Saint Lawrence	Pointe au Père (PAP)	5	66.3	0.4103 ± 0.3125	0.3820 ± 0.1378
	HR Crossing (HRC)	7	77	0.3775 ± 0.3010	0.3436 ± 0.1506
	Lower Barney's River (LBR)	8	78.4	0.3524 ± 0.2958	0.3249 ± 0.1526
Total		20			
Nova Scotia	Clam Harbour (CLA)	4	59	0.4350 ± 0.3149	0.4160 ± 0.1328
	Whynacht's Point (WHY)	4	60.8	0.4475 ± 0.3093	0.4212 ± 0.1263
	Digby (DGB)	4	64	0.4395 ± 0.3020	0.4072 ± 0.1320
Total		12			
Bay of Fundy	Minudie (MN)	4	63.8	0.4450 ± 0.3163	0.4184 ± 0.1304
	Grande Anse (GA)	3	56.1	0.5067 ± 0.3202	0.4669 ± 0.1157
	Daniels Flat (DF)	5	68	0.4154 ± 0.3057	0.3824 ± 0.1396
Total		12			
Gulf of Maine	Lubec (LBS)	3	59.9	0.5126 ± 0.3095	0.4676 ± 0.1152
	Machiasport (MAC)	2	44.7	0.5749 ± 0.3147	0.5697 ± 0.0823
	Falmouth (FAL)	3	56.8	0.4991 ± 0.3082	0.4625 ± 0.1175
	Audubon (AUD)	3	57.3	0.4851 ± 0.3085	0.4619 ± 0.1154
	Beach Avenue Maine (BAM)	5	68	0.4158 ± 0.3094	0.3795 ± 0.1426
Total		16			

### 3.3 Outlier detection

Using the results of the STRUCTURE analysis as a guide, we detected a total of 531 outliers from LOSITAN, and nine from BayeScan, between the Bay of Fundy and populations sampled elsewhere. Of these outliers, 92 from LOSITAN and all from BayeScan were putatively under directional selection. One BayeScan outlier was not found by LOSITAN, yielding a cumulative 93 putatively directionally selected outlier loci. A combination of all detected outliers from both datasets was used to filter the 2,583 SNPs into a conservatively neutral dataset consisting of 1,089 SNPs, each from a single contig to minimize bias caused by linkage disequilibrium.

### 3.4 Population structure

**Figure 2:**
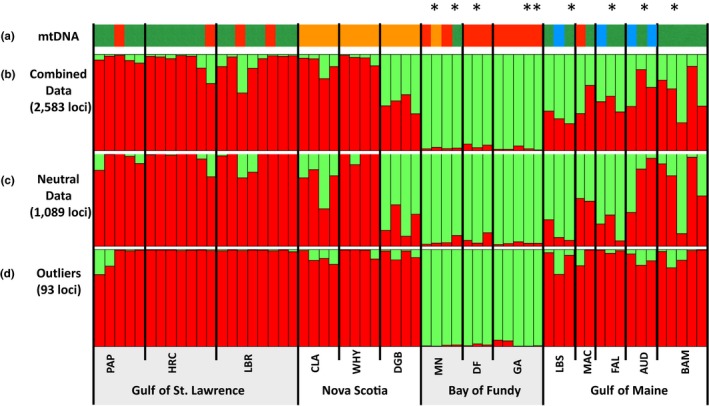
Bar plot of (a) mtDNA haplotype lineage (COI/COIII) of each individual used in the GBS analysis (colors similar to the pie charts in Figure 1) and STRUCTURE results for (b) combined, (c) neutral, and outlier SNPs from 60 individuals sampled from north to south along the coast. The horizontal axis indicates individuals grouped by populations within regions and the vertical axis indicates the posterior probability of assignment to the *k *=* *2 detected clusters. The mtDNA lineage of each individual marked with an asterisk was determined using 359 bp of COIII only (GenBank Accession numbers MF631028‐MF631036)

We analyzed the full (2,583 loci), neutral (1,089 loci), and outlier (93 loci) SNP datasets in STRUCTURE and used the method described by Evanno et al. (2005) to determine the most likely number of *k* clusters. The analysis of the LnP(D) for all three datasets indicated that *k* = 2 was the most likely number of clusters (Figures 2 and S1). For all three datasets, populations sampled within the Bay of Fundy were consistently assigned to the same cluster with admixture proportions ranging from 0.935 to 0.996. Similarly, the probability of assignment to the cluster consisting of Atlantic genotypes was consistently high for the GSL and coastal NS populations for all three datasets (Figure 2). However, admixture proportions for the populations sampled at or around the mouth of the Bay of Fundy (LBS; DGB) and south into the GOM varied depending on the data analyzed, with the outlier dataset providing the lowest admixture proportions with the Bay of Fundy genotypes, while more than half the individuals were assigned to BF cluster with admixture proportions >0.50 for the strictly neutral dataset.

AMOVA for all three datasets yielded significant fixation index values (*p *<* *.05). The full SNP dataset (2,583 loci) yielded a Φ_CT_ of 0.0122 between the two regions, indicating a small but significant differentiation between BF and elsewhere (Table 3). Φ_ST_ was similar at 0.0183, indicating that the majority of the variance (98.17%) was attributed to within population differentiation, with very little variance among populations within groups (Φ_SC_ = 0.0062). For the neutral SNP dataset, AMOVA results showed a small but significant amount of variation among groups (Φ_CT_ = 0.0057) or among populations within groups (Φ_SC_ = 0.0114). In contrast, results for the outlier SNP dataset showed a much greater amount of variation among regions (Φ_CT_ = 0.2144) and among populations within regions (Φ_SC_ = 0.0825; Table 3).

Patterns of pairwise *F*_ST_ between all 14 sampling sites for the full dataset and the outliers are broadly consistent with the STRUCTURE analysis and AMOVA (Table 2). With the exception of the sites sampled in Nova Scotia, pairwise *F*_ST_ values were generally small between populations within regions for both the full and outlier datasets (Table 2). Regional population structure was more pronounced in the northern part of the range, with pairwise *F*_ST_ values being relatively greater between populations sampled in GSL, NS, and BF (Table 2). Lower levels of population divergence were detected in the full dataset for comparisons between the inner Bay of Fundy and populations immediately outside the bay (LBS, DGB) and throughout the Gulf of Maine (*F*_st_ 0–0.0236; Table 2). However, consistently high genetic differentiation was detected between the BF and all other populations (*F*_ST_ = 0.1815–0.3456; Table 2).

**Table 2:** Pairwise *F*_ST_ values calculated by Arlequin 3.5.2.2 between each sampling site of *Macoma petalum* for the combined data (2,583 SNPs; above the diagonal) and the outlier loci (93 SNPs; below diagonal). The sites from each region are separated by a solid line; values in bold were significant at the 0.05 level after 10,100 permutations

	PAP	HRC	LBR	WHY	CLA	DGB	MN	GA	DF	LBS	MAC	FAL	AUD	BAM
PAP	–	**0.0048**	**0.0065**	**0.0030**	**0.0134**	**0.0138**	**0.0121**	**0.0259**	**0.0062**	**0.0194**	0.0009	**0.0014**	0	**0.0086**
HRC	**0.0873**	–	**0.0045**	**0.0096**	**0.0192**	**0.0163**	**0.0146**	**0.0281**	**0.0118**	**0.0134**	**0.0146**	**0.0091**	**0.0012**	**0.0105**
LBR	**0.0834**	0.0390	–	**0.0098**	**0.0212**	**0.0166**	**0.0175**	**0.0236**	**0.0061**	**0.0163**	**0.0178**	**0.0716**	**0.0045**	**0.0072**
WHY	**0.1216**	**0.1196**	**0.0863**	–	**0.0112**	**0.0111**	**0.0192**	**0.0283**	**0.0918**	**0.0122**	0.0058	**0.0036**	**0.0146**	**0.0272**
CLA	0.0189	**0.0767**	0	0.1098	–	**0.0128**	**0.0207**	**0.0294**	**0.0176**	**0.0172**	0.0231	**0.0127**	**0.0078**	**0.0316**
DGB	**0.1063**	**0.1003**	**0.0763**	0.0338	**0.1388**	–	0	**0.0146**	0.0045	0	0	0	0.0006	**0.0139**
MN	**0.2719**	**0.3163**	**0.3090**	**0.2515**	**0.3456**	**0.2366**	–	0	0	0	0.0005	0	0	0.0053
GA	**0.2173**	**0.2853**	**0.2854**	0.1864	**0.3192**	**0.1815**	0.0279	–	0	**0.0112**	**0.0122**	**0.0236**	**0.0090**	**0.0163**
DF	**0.2970**	**0.3379**	**0.3168**	**0.2551**	**0.3436**	**0.2672**	0.0717	**0.1594**	–	0	0.0015	0.0050	0	**0.0121**
LBS	**0.1307**	0.0797	0.0744	0.0695	0.1085	0.0506	**0.2200**	0.1912	**0.2379**	–	0	0	0	**0.0117**
MAC	0.0999	0.0643	0.0463	0.0691	0.1141	0	0.2468	0.2328	0.2864	0.0611	–	0	0	**0.0061**
FAL	0.0796	0.1024	0.0320	0.0835	0.0346	0	**0.2644**	0.2416	**0.2808**	0.0400	0	–	0	**0.0009**
AUD	0.1094	**0.1232**	0.0537	0.0209	0.1029	0	**0.2632**	0.2083	**0.2635**	0.0803	0	0.0226	–	0
BAM	**0.1003**	**0.1264**	**0.1053**	**0.1190**	**0.1652**	0.0722	**0.2767**	**0.2824**	**0.2902**	0.1050	0	0.0590	0.0714	–

**Figure 3:**
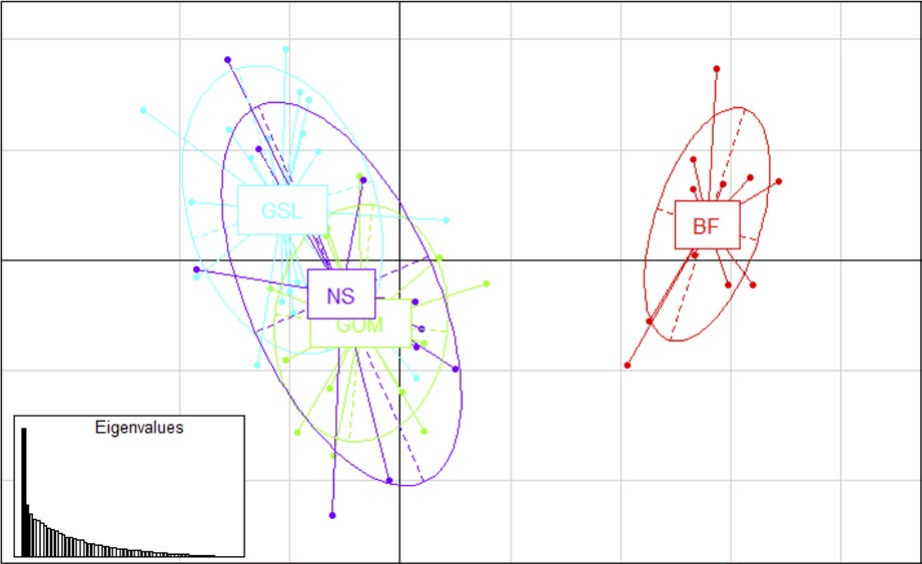
Graph of PCA using 93 putatively positively selected outliers between the Bay of Fundy (BF) and the other three regions: Gulf of Maine (GOM), Nova Scotia (NS), Gulf of Saint Lawrence (GSL). The first two principle components explain 22.7% of the total variation in the data

**Figure 4:**
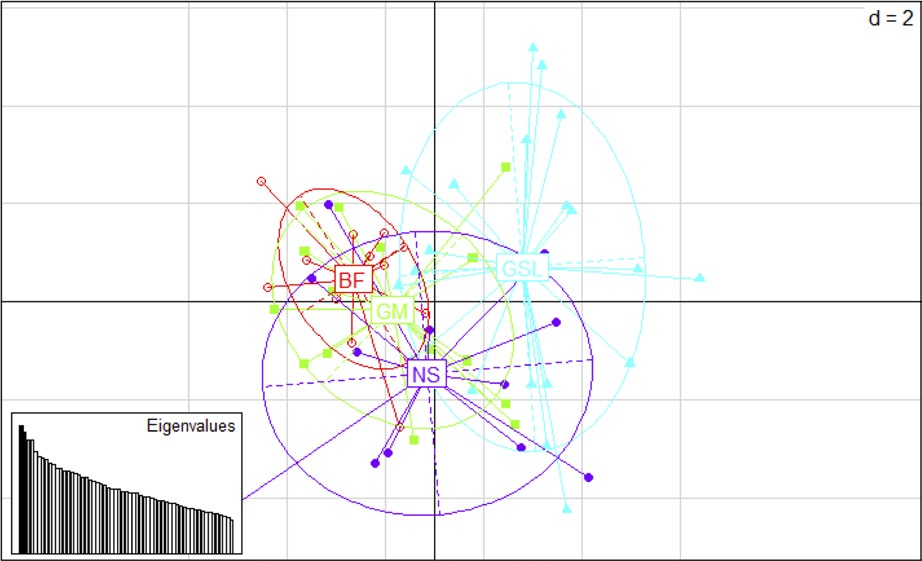
Graph of PCA using 1089 neutral loci. Individuals are marked by sampling region: Gulf of Maine (GOM), Bay of Fundy (BF), Nova Scotia (NS), and Gulf of Saint Lawrence (GSL). The first two principle components explain 6.37% of the total variation in the data

The results of PCA using the putative directionally selected outliers between BF and all other regions are shown in Figure 3. This outlier dataset clearly showed that the BF was separate from all other regions and that the GOM, GSL, and NS tended to group together. The first two principle components here explained 22.7% of the total variation. For the neutral dataset of 1,089 SNPs, the BF overlapped greatly with the GOM and NS regions and much less structure overall was present, although the GSL seemed to be less similar to the other three regions (Figure 4). The first two principle components of the neutral dataset explained only 6.37% of the total variation.

### 3.5 Outliers and evidence of selection

Outlier detection methods as well as BLASTX were used to identify genes putatively under selection and to determine potential targets of selection in *M. petalum* (Table 4). The 2,583 SNPs used for analyses were found on 1,415 unique contigs and singletons. Of these unique sequences, 439 returned a BLAST hit with an *e*‐value ≤10^−4^, 279 of these sequences additionally had a match using Interpro scan, and 231 returned a GO ID. The top hit BLAST results came from several species, including multiple hits from *Crassotrea gigas*, the Pacific Oyster, *Strongylocentrotus purpuratus*, the purple sea urchin, *Branchiostoma floridae*, the amphioxus, and *Tribolium castaneum*, the red flour beetle. Other hits came from arthropods, vertebrates, and from a gill symbiont. Ninety‐three outliers from 83 contigs and singletons putatively under directional selection were compiled from LOSITAN and BayeScan results between the Bay of Fundy and the other three regions. Of these outliers, 14 contigs returned a BLAST hit and had GO terms assigned (Table S1). From the consensus between LOSITAN and BayeScan, six of the putatively directionally selected outliers returned a BLAST hit. The six outlier sequences with BLAST results contained nine outlier SNPs and two neutral SNPs (Table 4). Of these, four contigs had a locus‐specific *F*_ST_ > 0.5 and two had GO terms assigned (Table 4). No alternative alleles were fixed between regions.

## 4 DISCUSSION

The recent advances of reduced representation next‐generation sequencing (NGS) permit the analysis of nuclear single nucleotide polymorphisms (SNPs) throughout the genome, providing both neutral and putatively selected markers (Baird et al., 2008; Elshire et al., 2011; Narum, Buerkle, Davey, Miller, & Hohenlohe, 2013; Peterson, Weber, Kay, Fisher, & Hoekstra, 2012; Poland, Brown, Sorrells, & Jannink, 2012). As we only consider SNPs in coding genes, our study provides the opportunity to assess the contributions of both genetic drift and natural selection to the maintenance of genetic divergence in a marine species for which barriers to dispersal are subtle. We detected a strong genetic break in the Atlantic population of *M. petalum* between the Bay of Fundy and the Gulf of Maine using putatively selected SNPs (outlier loci). Previous analysis of mtDNA in *M. petalum* showed hierarchical structure among four broad geographic regions (GOM, BF, NS, and GSL) for this species as well as the presence of a phylogeographic break at the Bay of Fundy (Einfeldt et al., 2017; Figures 1 and 2). While we failed to detect similar patterns of hierarchical structure among regions in our data, neutral SNPs showed a clear, but weak, pattern of isolation across the biogeographic break, suggesting some limitation in gene flow between the Bay of Fundy and Gulf of Maine. In contrast, analyses of outlier loci reveal a striking signal of isolation between the Bay of Fundy and the Gulf of Maine, suggesting that natural selection and local adaptation play a key role in driving genetic change among the regions. Outlier tests indicate that the allele frequencies at a subset of the SNPs appear to be driven by natural selection, in particular for genes whose functions include immunity (Table 4).

### 4.1 Population genetic structure

Our results are broadly consistent with analyses of mtDNA sampled from the same individuals (Einfeldt et al., 2017). We detected greater pairwise *F*_ST_ among the northern populations (GSL and NS) than those sampled in the Bay of Fundy and Gulf of Maine (Table 4), suggesting that higher levels of gene flow connect populations in the south. However, we failed to detect the hierarchical population structure in the mtDNA data because of the weak patterns of genetic differentiation among sample sites in the southern part of the range. Here, the magnitude of *F*_ST_ values in the SNP data is smaller, and majority of the comparisons between BF and GOM populations are not significantly different from zero. As populations of intertidal marine species including *M. petalum* recently expanded northward along the Atlantic coast following the LGM (e.g., Wares & Cunningham, 2001), and the coastal regions of the Bay of Fundy only recently became ice‐free within the last 13,000 years (Shaw et al., 2006), coalescent theory predicts incongruence between mitochondrial and nuclear loci because genetic drift has a greater impact on the lower effective population size of mtDNA. Thus, we expect greater divergence among populations of over shorter timescales and for relatively young postglacial populations such as *M. petalum*, and weak patterns observed in nDNA do not contradict the strong inferences of recent isolation and population divergence based on mtDNA.

Patterns of population connectivity throughout the study system, and in particular among the southern populations and those sampled in and around the Bay of Fundy, are broadly consistent with the expectations based on oceanic circulation and coastal current patterns. Limited gene flow caused by the current system in the GOM is driving genetic differentiation between the BOF and the GOM; however, these currents do not completely inhibit dispersal of planktonic particles (Pringle, Byers, He, Pappalardo, & Wares, 2017; Tilburg, McCartney, & Yund, 2012). The current system in the GOM consists of two coastal current regimes, the Eastern Maine Coastal Current (EMCC) and the Western Maine Coastal Current (WMCC; Figure 1). Both of these branches of the coastal current exhibit cyclonic circulation (Pettigrew et al., 2005). More importantly, the EMCC cycles counterclockwise from the mouth of the Bay of Fundy along the eastern coast of Maine to the Penobscot Bay (Pettigrew et al., 2005). Circular currents are known to prevent mixing (White et al., 2010). Therefore, the EMCC likely minimizes gene flow between the Bay of Fundy and the Gulf of Maine, as seen in *C. volutator*,* H. diversicolor*, and *T. obsoleta* (Einfeldt & Addison, 2013; Einfeldt et al., 2014, 2017). Tilburg et al. (2012) showed that the EMCC can be a barrier to larval dispersal in the absence of wind‐driven waves, but does not completely prevent dispersal. Comparison of mtDNA data from Einfeldt et al. (2017) with the nuclear SNPs reported here are consistent with this low level of dispersal into the BF, as unique mtNDA haplotypes found along the eastern coast of Nova Scotia are found at low frequencies within the BF populations. However, the near absence of admixture of coastal genotypes in BF populations suggests that gene flow is low across the nuclear genome as a result of limited gene flow and strong natural selection.

In addition to consistency among empirical population genetic studies of marine species, our results are in good alignment with models of propagule transport and range boundaries in coastal species (e.g., Altman, Robinson, Pringle, Byers, & Wares, 2013; Pappalardo, Pringle, Wares, & Byers, 2015; Pringle et al., 2017). Pringle et al. (2017) modeled the dispersal and local recruitment of larvae in the Bay of Fundy and at sites along the coasts of the Gulf of Maine. Species for which planktonic dispersal was 15 days exhibited a much greater proportion of retention of larvae in the Bay of Fundy than along coastal GOM. Our data are consistent with this model as there was low admixture across all loci within the BF, but the average admixture proportions of populations in the GOM were moderate (combined loci 0.35–0.66; neutral loci 0.32–0.86; Figure 2) which suggests relatively high connectivity with the BF populations. In addition, the mtNDA results indicate a general southern dispersal pattern, particularly when considering the unique haplotpyes from coastal NS (Figure 1; Einfeldt et al., 2017). However, the absence of similar patterns of asymmetric gene flow of outlier loci out of the Bay of Fundy and into Digby or south along the coast of the GOM suggests that gene flow and drift continue to balance natural selection and local adaptation across this boundary.

### 4.2 Putative selection in protein‐coding genes

In addition to reduced gene flow and genetic drift, our results indicate that natural selection may also be driving differentiation between the Bay of Fundy, the Gulf of Maine, the Gulf of Saint Lawrence, and the Nova Scotia populations of *M. petalum*. Nuclear SNPs mapped to the *M. balthica* partial transcriptome showed evidence of directional selection at 93 putatively selected loci on 83 contigs (Figure 3, Table 4). The signal of differentiation between the BF and the GOM was present for neutral loci (Figure 2), but is stronger for putatively selected loci, as visualized in the PCAs of neutral‐only and outlier‐only loci (Figures 3 and 4). Additionally, *Φ*_CT_ among regions was 20 times greater for outlier SNPs than for neutral SNPs (Table 3).

**Table 3:** Variance partitioned between populations of *Macoma petalum* as determined by a standard analysis of molecular variance (AMOVA) using Arlequin 3.5.2.2. Analyses were performed with all 2,583 single nucleotide polymorphisms (SNPs), 1,089 neutral SNPs, and 93 outlier SNPs with populations grouped into two sampling regions: (the Bay of Fundy) and (the Gulf of Saint Lawrence, Nova Scotia, and the Gulf of Maine)

	Source of variation	Degrees of freedom	Sum of squares	Variance components	Percentage of variation	Fixation index	*p*‐Value
Full dataset (2583 loci)	Among groups	1	513.2	4.16	1.22	Φ_CT_ = 0.0122	<.0001
	Among populations within groups	12	4236.4	2.102	0.62	Φ_SC_ = 0.0062	<.0001
	Within populations	106	35535.8	335.2	98.17	Φ_ST_ = 0.0183	<.0001
Neutral dataset (1089 loci)	Among groups	1	195.3	0.85	0.56	Φ_CT_ = 0.0057	.0115
	Among populations within groups	12	1947.8	1.704	1.13	Φ_SC_ = 0.0114	<.0001
	Within populations	106	15675.9	147.9	98.3	Φ_ST_ = 0.0170	<.0001
Outlier dataset (93 loci)	Among groups	1	120.8	2.721	21.44	Φ_CT_ = 0.2144	<.0001
	Among populations within groups	12	193.3	0.822	6.48	Φ_SC_ = 0.0825	<.0001
	Within populations	106	969.5	9.146	72.08	Φ_ST_ = 0.2792	<.0001

No alternative alleles were fixed between regions; therefore, allele frequencies were used to investigate the differential presence of the alternate allele between regions. Due to the nature of reduced representation genome sampling with restriction enzymes, wherein null alleles are excluded, and that only SNPs are considered here, allele frequencies are biased to homozygosity (Arnold, Corbett‐Detig, Hartl, & Bomblies, 2013; Davey et al., 2013; Puritz et al., 2014). This bias is the result of polymorphisms in the restriction enzyme cut sites being excluded along with structural variants. Therefore, actual frequencies should be considered with caution, and the trend between regions should be the main focus.

The outliers with BLAST results had functions generally related to immunity. These results include asialoglycoprotein receptor subunit 2 (ASGPR2) and 1,2‐dihydroxy‐3‐keto‐5‐methylthiopentene dioxygenase (membrane‐type 1 matrix metalloproteinase cytoplasmic tail binding protein‐1 or MTCBP‐1). Two top BLAST hits represented predicted uncharacterized proteins, and two were related to transposable elements. Frequencies of the alternative and the reference alleles are available in Table 4.

**Table 4:** Top BLAST hits of outliers putatively under positive selection as detected by both BayeScan and LOSITAN between sampling regions for *Macoma petalum*. A single asterisk indicates that the sequence has a locus‐specific *F*_ST_ > 0.5 for the dataset partitioned into two regions

Outlier contig	Number of outlier SNPs (Total number of SNPs)	Frequency of the outlier alternative allele(s) in BF	Frequency of the outlier alternative allele(s) in GOM, GSL, and NS	Top BLAST hit name	*e*‐Value	Accession	Top BLAST hit organism	GO ID	GO term	GO category
Contig_1482*	1 (2)	0.92	0.35	PREDICTED: uncharacterized protein LOC105346799	4.00E‐18	KHF24562.1	*Solemya velum* (Atlantic awning clam) gill symbiont	–	–	–
Contig_7314*	4 (5)	0.33 (×3), 0.5	0.06, 0.05, 0.06	1,2‐dihydroxy‐3‐keto‐5‐methylthiopentene dioxygenase (MTCBP‐1)	1.20E‐57	EKC28806.1	*Crassotrea gigas* (Pacific oyster)	GO:0005506 GO:0005634	Iron ion binding Nucleus	MF CC
Contig_9429*	1	0.42	0.07	PREDICTED: uncharacterized protein LOC106170666	4.90E‐23	–	*Lingula anatina* (Brachiopod)	–	–	–
Singleton_38180	1	0.42	0.08	Transposable element Tcb1 transposase	2.00E‐38	CDQ90832.1	*Oncorhynchus mykiss* (Rainbow trout)	–	–	–
Singleton_189054*	1	0.33	0.03	RNA‐directed DNA polymerase from mobile element jockey‐like	1.00E‐30	AAA49022.1	*Gallus gallus* (Chicken)	GO:0003824 GO:0044237	Catalytic activity Cellular metabolic process	MF BP
Singleton_137612	1	0	0.34 0.55 (GSL)	Asialoglycoprotein receptor 2 (ASGPR2)	1.40E‐06	EEN61059.1	*Branchiostoma floridae* (Florida lancelet)	–	–	–

BP, biological process; MF, molecular function; CC, cellular component.

ASGPR2 had the highest frequency of the alternative allele in the GSL. The frequency of the alternative allele in BF was 0, although only 12 individuals were sampled from BF so it is possible that this allele occurs at low frequencies in BF as the alternative allele was found in both GOM and NS. ASGPR, formed by subunits ASGPR1 and ASGPR2, has been associated with immune response in shrimp against virions and bacterial infections (Wongpanya, Aoki, Hirono, Yasuike, & Tassanakajon, 2007).

The five remaining sequences all had a differentially high proportion of the alternative allele in the BF. The contig that matched to MTCBP‐1 contained five SNPs, four of which were outliers. MCTBP‐1 plays a role in the inhibition of promoting of tumor cell migration and invasion caused by membrane‐type 1 matrix metalloproteinase (MT1‐MMP/MMP‐14; Stipanuk, Simmons, Karplus, & Dominey, 2011). Additionally, a truncated form of MCTBP‐1 allows the hepatitis C virus to replicate in nonpermissive cell lines (Yeh, Lai, Chen, Chu, & Liaw, 2001). Overall, ASGPR2 and MTCBP‐1 may be under selection in *M. petalum* against unique infective agents among the sampled regions driving the variation in allele frequencies at these loci.

Two of the outlier contigs represent predicted, uncharacterized proteins. One of these uncharacterized proteins had two SNPs, only one of which was under putative selection with a locus‐specific *F*_ST_ > 0.5 (Table 4). The top BLAST hit organism for this protein was from the *Solemya velum*, the littoral‐dwelling Atlantic awning clam. Gamma proteobacterial gill symbionts are chemoautotrophs that live intracellularly within the gills of clams in species‐specific interactions (Eisen, Smith, & Cavanaugh, 1992). *Macoma petalum* is unlikely to host such symbionts as this species possesses siphons. The gills of several species of siphon‐possessing bivalves were examined and no bacteriocytes have been observed to date, possibly because the siphons of such species provide irrigation of the pallial cavities, removing sulfides, which are the metabolic substrate for these chemoautotrophs (Le Pennec, Beninger, & Herry, 1995). Possible causes for the presence of a gill symbiont protein include contamination of both the *M. petalum* GBS dataset and the *M. balthica* partial transcriptome, or that this protein is indeed part of the *Macoma* genome. Whether this putative protein is part of the *Macoma* genome is difficult to determine without knowing the function or type of protein, although multiple BLAST hits were found from the *S. velum* gill symbiont.

The other predicted uncharacterized protein originates from *Lingula anatina*, a brachiopod. Interpro scan results show that this protein contains an aspartic peptidase domain. Aspartic peptidases occur in a wide range of organisms, including vertebrates, fungi, plants, protozoa, bacteria, archea, retroviruses, and plant viruses with a wide range of functions, from gastric digestion, intracellular protein digestion to specific processing of precursor proteins (Davies, 1990; Rao, Erickson, & Wlodawer, 1991). Therefore, the activity of this putative protein in *M. petalum* likely involves protein digestion or processing of precursor proteins and may be important for a wide range of functions.

The final two outliers were both related to transposable elements. The transposable element Tcb1 transposase was a strong outlier with locus‐specific *F*_ST_ > 0.5. The Tcb1 transposon is part of the mariner‐Tc1 superfamily of transposons, which occur in plant, bacterial, invertebrate, and vertebrate genomes (Brownlie, Johnson, & Whyard, 2005; Ketting, Fischer, & Plasterk, 1997; Plasterk, 1991; Van Luenen & Plasterk, 1994). The final outlier sequence with a BLAST result was a eukaryotic RNA‐directed DNA polymerase from a *jockey*‐like mobile element. Found in *Drosophila*,* jockey* is a retrotransposon similar to mammalian LINEs (long interspersed elements), which are widespread throughout eukaryotic genomes (Ivanov, Melnikov, Siunov, Fodor, & Ilyin, 1991). The *jockey*‐like retrotransposons account for the formation of many pseudogenes (Bebikhov, Postnoc, & Nikinenko, 1998; Rashkova, Karam, & Pardue, 2002). Transposable elements are biased to regions of the genome in which reduced rates of recombination occur (Dolgin & Charlesworth, 2008; Rizzon, Marais, Gouy, & Biemont, 2002). Therefore, it is likely that the Tcb1 transposase and the RNA‐directed DNA polymerase from a *jockey*‐like mobile element are not themselves experiencing selective pressures, but that they are linked to loci that are under putative selection in *M. petalum*.

A major limitation of using reduced representation genomic methods with restriction enzymes is exclusion of some variable sites. Mutations in the enzyme cut sites themselves would result in an alternate allele being unknowingly discarded (Arnold et al., 2013; Davey et al., 2013; Puritz et al., 2014). In addition, we only used SNPs to investigate patterns of genetic differentiation when other variable sites, such as indels, are known to be informative in a variety of organisms and for a variety of purposes (Murphy, Pringle, Crider, Springer, & Miller, 2007; Soltis et al., 1997). This study seeks to explore the genomics of *M. petalum* in the Atlantic and propose some correlations between observed phylogeographic patterns and putative selection, not claim outright the causation of these patterns or to rigorously determine all genes putatively under selection.

The outliers detected and described above do not show a clear, single‐factor differentially affecting sample sites in the BF compared to the GSL, GOM, or NS regions. However, many outlier sequences did not have BLAST results, and may represent important proteins that have not been adequately described in bivalves to date. Additionally, no alternative alleles were differentially fixed between regions. Regardless, the differentiation between the BF and the other regions is clear, and is most likely caused by a mix of neutral factors, such as the EMCC limiting gene flow and facilitating genetic drift, and natural selection. Two of the outliers with BLAST results had functions related to immunity. Selection for immunity could be in response to parasites, infectious bacteria, viruses, or cancerous diseases as in *Mya arenaria* (Metzger, Reinisch, Sherry, & Goff, 2015). Pollutants also pose a risk to marine bivalves and can have effects on their immune system (Giron‐Perez, 2010). The Bay of Fundy is home to aquaculture sites for salmon, which increase levels of xenobiotic compounds in surrounding areas. Poor circulation due to the Gulf of Maine Coastal Currents, which are suspected to prevent dispersal in some marine invertebrates (Einfeldt & Addison, 2013; Einfeldt et al., 2014, 2017), suggests that these compounds may not be flushed into the open ocean with the tide, but are possibly retained and concentrated in the Bay of Fundy. Xenobiotic compounds found at high levels around aquaculture sites include polycyclic aromatic hydrocarbons (PAHs) which are carcinogenic and mutagenic (Nagai, Kano, Funasaka, & Nakamuro, 2002), polychlorinated biphenyls (PCBs) which are carcinogenic (Faroon, Keith, Jones, & de Rosa, 2001), and chlorinated pesticides (Hellou, Haya, Steller, & Burridge, 2005). Toxic metals have also been detected at high levels in BF sediments, including mercury, arsenic, lead, and vanadium (Hung & Chmura, 2006, 2007). Bivalves, being filter feeders, may accumulate high concentrations of pathogens, heavy metals, or other pollutants in their tissues (Song, Wang, Qiu, & Zhang, 2010). Therefore, differentiation of *M. petalum* in the Bay of Fundy could be caused by selection for immunity to such pollutants and their immunological effects, or other disease causing agents, as well as limited gene flow from the EMCC.

## 5 CONCLUSION

*Macoma petalum* shows a distinct genotypic difference between the Bay of Fundy and the other three regions examined, Nova Scotia, the Gulf of Maine, and the Gulf of Saint Lawrence. While previous work on *Macoma petalum* identified a genetic discontinuity at or near the mouth of the Bay of Fundy (Einfeldt et al., 2017), this study extends that finding by demonstrating weak differentiation at neutral loci and strong natural selection across this range boundary. Our results show that divergence across this break is driven by both limited gene flow resulting from circular currents that inhibit the mixing of the Gulf of Maine and the Bay of Fundy waters and local selective pressures on populations within the Bay of Fundy mudflats. The fact that the Bay of Fundy genotype is found in the surrounding populations in the Gulf of Maine and in Digby, Nova Scotia, but that the genotypes from these other populations are not found in the Bay Fundy, suggests that cyclonic currents result in asymmetric gene flow out of, but not into, the Bay of Fundy. In addition, outlier loci detected between the Bay Fundy and all other regions suggest that natural selection may be driving local adaptation within the Bay of Fundy. The role of these outliers in selection is not clear; however, there may be differential selection due to immunological pressures, such as pathogens or pollution. This study presents a first step in examining the putative presence of directional selection of populations of *M. petalum* in the Bay of Fundy.

## CONFLICT OF INTEREST

None declared.

## AUTHOR CONTRIBUTIONS

SLM, JHK, and JAA conceived and designed the study; SLM and JHK performed the experiments and collected the data; SLM, JHK, and JAA analyzed and interpreted the data; JAA contributed resources; SLM and JAA drafted the manuscript; and SLM, JHK, and JAA edited and revised the final draft.
