# Supplementary material for: Genotype by sequencing identifies natural selection as a driver of intraspecific divergence in Atlantic populations of the high dispersal marine invertebrate, Macoma petalum
Source: Ecol Evol. 2017 Sep 3;7(19):8058–72. doi: 10.1002/ece3.3332 (PMC5632645; doi:10.1002/ece3.3332)
Supplement: Supplementary file 1 [file ECE3-7-8058-s001.docx]

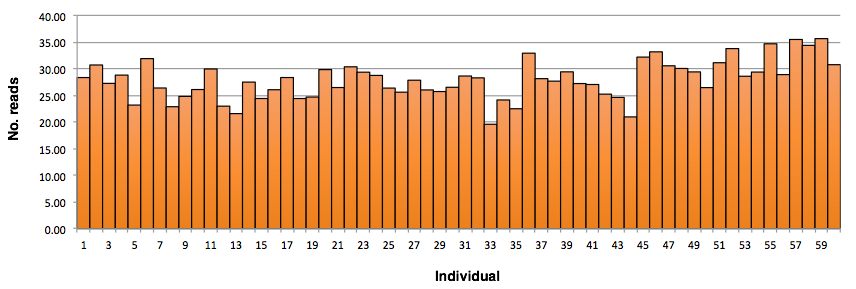


Figure S1. Average depth of heterozygous SNPs (no. of heterozygous SNP-detected reads / no. of heterozygous SNPs) for each individual sampled (arranged from north to south, see Structure figure in main text).


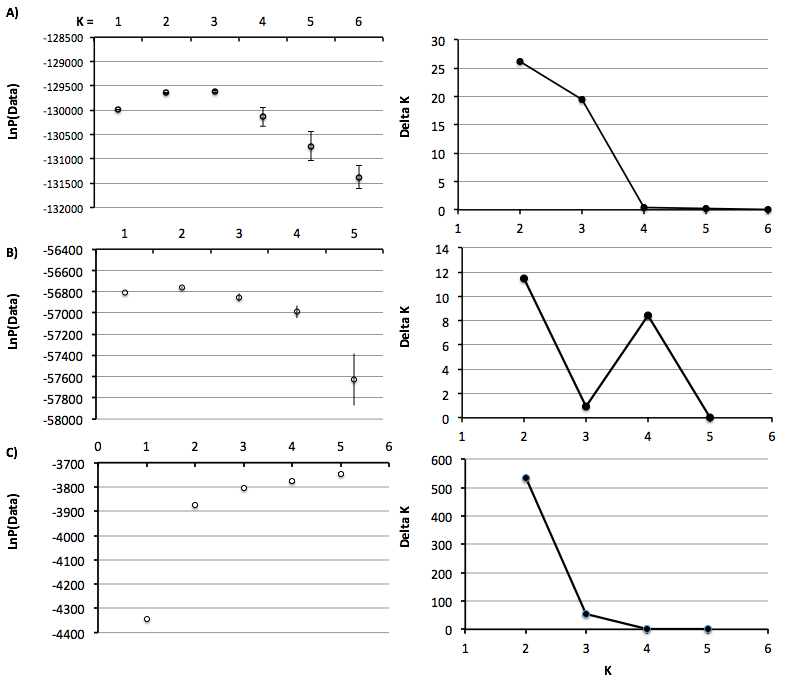


Figure S2. STRUCTURE (Pritchard *et al.*, 2000) results fro *Macoma petalum* using A) 2583 SNPs, B) 1089 neutral SNPs, and C) 93 putative outlier SNPs. Left panels plot the Mean Log probability data ((LnP(D); error bars ± SD) as a function of *k* (number of clusters) from 10 replicates per *k*; Right panels plot Delta-K of LnP(D) as a function of *k* (Evanno *et al.*, 2005; Dent and vonHoldt, 2012).

Table T1: Outliers annotated with gene ontology (GO) terms in a category of biological process (BP), molecular function (MF), and cellular component (CC)

| Sequence ID | Sequence Description | GO ID | GO Term | GO Category |
| --- | --- | --- | --- | --- |
| Contig_759 | Quinone oxidoreductase isoform X2 | GO:0008270  GO:0016491  GO:0055114 | Zinc ion binding  Oxidoreductase activity  Oxidation-reduction process | MF  MF  BP |
| Contig_1390 | DNA polymerase | GO:0044260  GO:0090304 | Cellular macromolecule metabolic process  Nucleic acid metabolic process | BP  BP |
| Contig_1630 | Uncharacterized transposon-derived | GO:0003676  GO:0015074 | Nucleic acid binding  DNA integration | MF  BP |
| Contig_4219 | Malate cytoplasmic | GO:0002230  GO:0005615  GO:0005739  GO:0005813  GO:0005829  GO:0005975  GO:0006090  GO:0006108  GO:0019643  GO:0030060  GO:0043209  GO:0046487  GO:0070062  GO:0098779  GO:0098792 | Positive regulation of defense response to virus by host  Extracellular space  Mitochondrion  Centrosome  Cytosol  Carbohydrate metabolic process  Pyruvate metabolic process  Malate metabolic process  Reductive tricarboxylic acid cycle  L-malate dehydrogenase activity  Myelin sheath  Glyoxylate metabolic process  Extracellular exosome  Positive regulation of macromitophagy in response to mitochondrial depolarization  Xenophagy | BP  CC  CC  CC  CC  BP  BP  BP  BP  MF  CC  BP  CC  BP  BP |
| Contig_4517 | Retrovirus-related Pol poly from transposon 412 | GO:0003676  GO:0015074  GO:0046872 | Nucleic acid binding  DNA integration  Metal ion binding | MF  BP  MF |
| Contig_6667 | DNA replication complex GINS PSF3 | GO:0005634  GO:0006260 | Nucleus  DNA replication | CC  BP |
| Contig_7314 | 1,2-dihydroxy-3-keto-5-methylthiopentene dioxygenase | GO:0005506  GO:0005634  GO:0005737  GO:0010309  GO:0019509  GO:0055114 | Iron ion binding  Nucleus  Cytoplasm  Acireductone dioxygenase [iron(II)-requiring] activity  L-methionine salvage from methylthioadenosine  Oxidation-reduction process | MF  CC  CC  MF  BP  BP |

**Literature cited:**

Earl, DA, vonHoldt, BM (2012) STRUCTURE HARVESTER: a website and program for visualizing STRUCTURE output and implementing the Evanno method. Conservation Genetics Resources vol. 4 (2) pp. 359-361 doi: 10.1007/s12686-011-9548-7

Evanno G, Regnaut S, Goudet J (2005) Detecting the number of clusters of individuals using the software STRUCTURE: a simulation study. Mol Ecol 14:2611–2620

Pritchard JK, Stephens M, Donnelly P (2000) Inference of population structure using multilocus genotype data. Genetics 155:945–959
